# Investigating the cross-lingual translatability of VerbNet-style classification

**DOI:** 10.1007/s10579-017-9403-x

**Published:** 2017-10-20

**Authors:** Olga Majewska, Ivan Vulić, Diana McCarthy, Yan Huang, Akira Murakami, Veronika Laippala, Anna Korhonen

**Affiliations:** 1 Language Technology Lab (LTL), Department of Theoretical and Applied Linguistics (DTAL), University of Cambridge, 9 West Road, Cambridge, CB3 9DP UK; 2 Department of French Studies, University of Turku, 20014 Turku, Finland

**Keywords:** VerbNet, Multilingual NLP, Levin verb classes, Lexical-semantic classification

## Abstract

VerbNet—the most extensive online verb lexicon currently available for English—has proved useful in supporting a variety of NLP tasks. However, its exploitation in multilingual NLP has been limited by the fact that such classifications are available for few languages only. Since manual development of VerbNet is a major undertaking, researchers have recently translated VerbNet classes from English to other languages. However, no systematic investigation has been conducted into the applicability and accuracy of such a translation approach across different, typologically diverse languages. Our study is aimed at filling this gap. We develop a systematic method for translation of VerbNet classes from English to other languages which we first apply to Polish and subsequently to Croatian, Mandarin, Japanese, Italian, and Finnish. Our results on Polish demonstrate high translatability with all the classes (96% of English member verbs successfully translated into Polish) and strong inter-annotator agreement, revealing a promising degree of overlap in the resultant classifications. The results on other languages are equally promising. This demonstrates that VerbNet classes have strong cross-lingual potential and the proposed method could be applied to obtain gold standards for automatic verb classification in different languages. We make our annotation guidelines and the six language-specific verb classifications available with this paper.

**Electronic supplementary material:**

The online version of this article (doi:10.1007/s10579-017-9403-x) contains supplementary material, which is available to authorized users.

## Introduction

Lexical resources have played an instrumental role in supporting many NLP applications (Grishman et al. 1994; Miller 1995; Baker et al. 1998; Hovy 2006). Incorporating a vast range of linguistic properties, they allow one to abstract away from individual words and infer information about their behaviour, proving helpful in NLP tasks where data sparseness is a problem. Rich lexical resources are particularly important for verbs that typically act as main predicates of sentences and carry key syntactic-semantic information for language understanding. For verbs, one of the richest lexical resources currently available is VerbNet (Kipper et al. 2000; Kipper Schuler 2005). Capturing generalisations about morpho-syntactic and semantic properties of verbs, VerbNet has been used to support a variety of NLP tasks, including semantic role labeling, computational lexicography, information extraction, and question-answering (e.g., Swier and Stevenson 2004; Brown and Palmer 2012; Crouch and King 2005). Its predictive capacity, thanks to which systems can perform better on unseen vocabulary by extrapolating from individual words to classes, coupled with its ability to render higher level abstractions like semantic or syntactic features (Kipper et al. 2006), makes VerbNet a useful tool that can help overcome the issue of insufficient empirical data in machine translation, parsing, or word sense disambiguation (Aziz and Specia 2011; Shi and Mihalcea 2005; Bailey et al. 2015; Dang 2004; Kawahara and Palmer 2014; Windisch Brown et al. 2011).

Based on the work of Beth Levin (1993), VerbNet-style classification has been argued to have a strong cross-lingual element, and interrelatedness of verbs’ semantics and syntactic behaviour is believed to be universal across languages (Jackendoff 1992; Levin 1993). This, together with the potential benefit in multilingual NLP, has motivated recent work towards development of VerbNets for other languages, including Spanish and Catalan (Aparicio et al. 2008), Czech (Pala and Horák 2008), Mandarin (Liu and Chiang 2008), and Italian (Busso and Lenci 2016), however, similar resources are still only available for a small group of languages. Manual development of such taxonomies using Levin’s methodology is extremely time consuming. A cost-effective alternative that has been used to support both the manual development of VerbNets (e.g., Pala and Horák 2008) as well as creation of gold standards for (semi-)automatic verb classification (Sun et al. 2010; Scarton et al. 2014) is translation of VerbNet classes from English to other languages.

While the translation approach has a theoretical justification in linguistic literature and whilst it was employed extensively in the related (yet easier, as purely semantic) task of the translation of WordNet (Fellbaum 1998) into other languages (Vossen 1998; Isahara et al. 2008; Lindén and Carlson 2010), no empirical investigation of the translatability of VerbNet-style classification across languages has yet been conducted.

Our study aims to address this problem. We take a subset of VerbNet used in previous work (Sun et al. 2010) and investigate its translatability to a set of six typologically diverse languages: Polish, Croatian, Italian, Mandarin, Japanese, and Finnish. We introduce a systematic language-independent method and guidelines for the translation of the classes. These were first applied to Polish to investigate the expertise needed for translation as well as the role of language-specific information in the process. An experiment with six native speakers of Polish is reported which shows that the translation process is reliable: there is a high degree of translatability ($\approx$ 96% of member verbs directly translated) and high inter-annotator agreement (IAA) between the translators. The same methodology was then applied to the five other languages. We show that while certain language-specific adjustments were needed to adequately translate verbs and examine their subcategorisation properties, and while, in some cases, language-specific considerations could be used to enhance the classification further, the basic language-independent steps in the procedure are applicable to all the languages studied.

This first systematic investigation of translatability of VerbNet classes adds empirical support to the theoretical arguments about the cross-lingual applicability of Levin-style classes and shows that translation can be used to create highly accurate classifications, even for languages that are typologically distant from English. The guidelines and lexical resources are made available as supplementary online material with this paper (Online Resource 1 and 2).

The rest of the paper is organized as follows. Section 2 discusses the linguistic hypothesis and theoretical justification underlying Levin’s taxonomy and provides an overview of the architecture of VerbNet and related research, including recent cross-lingual work. Section 3 presents the method adopted for translating verb classes and its evaluation on Polish, chosen as the development language, and includes an analysis of inter-annotator agreement and challenges to the translation approach. In Sect. 4 the results of the multilingual experiment are presented in which the procedure described in Sect. 3 is applied to Croatian, Italian, Mandarin, Japanese, and Finnish, followed by the evaluation of an alternative automatic approach against our manual method and discussion of the impact of cross-linguistic variation on VerbNet translatability. Finally, in Sect. 5 we present conclusions and directions for future work.

## Levin classes and VerbNet

The basic classes in VerbNet (e.g., the class PUT for verbs such as *place*, *position* and *arrange*) are based on Levin’s (1993) verb classification. The classification captures the interrelatedness of verb behaviour and meaning in terms of systematic variations in the syntactic expression of verbal arguments called *diathesis alternations*, represented as sets of alternating verb frames that are related with the same or similar meaning. One example of such regular alternation in argument structure realisation is the so-called *middle* alternation (1), in which a transitive frame (a) alternates with a middle construction (b), where the patient or theme of a verb (its logical object) is realised as the subject. Native speakers of English intuitively know which verbs participate in this alternation and which do not:

**Table:** 

1)	a)	Sara broke the porcelain saucer.	2)	a)	The cyclist hit the gatepost.
	b)	Porcelain saucers break easily.		b)	*Gateposts hit easily.

Levin associates the speakers’ ability to make such judgments with a verb’s meaning: verbs patterning together with regard to diathesis alternations display shared meaning components. Conversely, the alternation behaviour of verbs can be largely predicted from their meaning. Guided by these theoretical assumptions, Levin manually classified 3024 English verbs (4186 senses), using 79 diathesis alternations as the primary selection criteria, also considering verb morphology, subcategorisation properties and extended verb meanings. The resultant taxonomy—the most widely used English resource of this kind in NLP—comprises 48 broad and 192 fine-grained subclasses, each characterised by a set of relevant alternations in which member verbs can participate.

VerbNet (Kipper Schuler 2005), a large hierarchical domain-independent, broad-coverage verb lexicon, employs and extends Levin classes, providing fine-grained syntactic and semantic information for them. The verbs are grouped into classes based on their shared meaning components and syntactic behaviour, defined in terms of their participation in diathesis alternations. Each class in this taxonomy is characterized by its member verbs, syntactic frames, semantic predicates and typical verb arguments. VerbNet employs thematic roles to represent verbal arguments and help disambiguate between classes sharing similar syntactic frames, and selectional restrictions to constrain the types of thematic roles possible for the verb’s arguments. The syntactic-semantic information displayed for each class is strictly monotonic: a given subclass inherits all the properties listed for its parent class, and includes additional information applicable specifically to its member verbs, for example further selectional restrictions or diathesis alternations. The monotonicity of the lexicon, as well as its hierarchical design, make it easier to integrate with other lexical databases. What is more, the taxonomy is characterised by different degrees of granularity, which is an important feature considering that the level of granularity which different NLP applications require varies from task to task.

The lexicon has mappings to a number of other verb resources, such as WordNet, FrameNet (Baker et al. 1998), or PropBank (Palmer et al. 2005) through the sets of mappings created by the SemLink project initiative (Loper et al. 2007; Palmer 2009). VerbNet has since been extended and constitutes the most extensive Levin-style classification of English verbs (Kipper et al. 2006; Bonial et al. 2013), containing 4402 unique verbs organised in 273 fine-grained classes (giving rise to over 6300 verb+class types), which has been used to benefit numerous NLP tasks in English (Korhonen 2002; Swier and Stevenson 2004, inter alia).

However, the classification system and the subsequent benefits for NLP should not be limited to English. Similar interrelatedness of syntactic behaviour and meaning of verbs can be found across languages, and despite discrepancies between particular verb and alternation inventories, the basic meaning components of verb classes have been argued to be cross-linguistically valid (Jackendoff 1992). For example, Levin (1993) notes that verbs in Warlpiri manifest analogous behaviour to English with respect to the conative alternation^1^ (it is found with *hit*-type and *cut*-type verbs, but not *break*-type and *touch*-type verbs). In Polish verbs pattern like English verbs in terms of the middle construction:

**Table:** 

3)	Porcelanowe	spodki	łatwo się	tłuką.
	porcelain.NOM.PL saucer.NOM.PL easily REFL break.3PL			
	‘Porcelain saucers break easily.’

**Table:** 

4)*	Słupy	łatwo się	uderzają.
	post.NOM.PL easily REFL hit.3PL		
	‘Posts hit easily.’		

The stipulated cross-linguistic validity of Levin classification not only reinforces the hypothesis lying at its core, but also has important implications for the creation of VerbNet style resources to support NLP in other languages. Yet few languages boast Levin-style classifications and resources similar to the English VerbNet. Some of these have been developed manually from scratch, aiming to capture properties specific to the language in question, e.g., resources for Spanish and Catalan (Aparicio et al. 2008), Czech (Pala and Horák 2008), and Mandarin (Liu and Chiang 2008). Others have been created (semi-)automatically, using already existing resources and aiming to reduce the time-expense involved, e.g., for Brazilian Portuguese (Scarton and Aluısio 2012), French (Pradet et al. 2014), and Croatian (Mikelić Preradović and Boras 2013). Also fully automatic methods have been used, e.g., for French (Sun et al. 2010) and Brazilian Portuguese (Scarton et al. 2014). However, this work has been limited to a small number of languages. The translation approach explored by some of these works could, if proven accurate, greatly facilitate resource creation and subsequent exploitation of Levin style classes cross-lingually. This would also help in evaluation of automatic approaches which have the potential to extend the coverage of such resources.

## Translation of verb classes: method and evaluation on Polish

We adopt the basic method of Sun et al. (2010) for translating VerbNet classes and improve it further to ensure maximum quality and consistency among translators. We chose Polish as the development language for the method and translation guidelines, to assess (i) the need for language-specific information and (ii) the expertise needed for translation. The translation task was first completed and the translation guidelines developed by a Polish native speaker with linguistics training (Online Resource 1). Following the pilot translation, five other native speakers of Polish (two with and three without linguistics training) independently performed the translation task according to the guidelines. They provided responses to a questionnaire (Table 1), aimed at eliciting quantifiable information about particular stages of the task, in order to supply a more objective measure of cross-lingual translatability of verb classes.

### Method and procedure

The classification chosen for translation was the VerbNet data used by Sun et al. (2008) and previously translated from English into French (Sun et al. 2010) and Brazilian Portuguese (Scarton et al. 2014). This resource includes 17 fine-grained classes sampled from different parts of the VerbNet taxonomy, with 12 member verbs each (e.g., ‘9.1 PUT’ class: *bury, place, install, put, mount, deposit, position, set, situate, immerse, insert, stash*).

The method had two main stages: (i) translation of the 12 member verbs from each class into the target language, (ii) selection and elimination of thus obtained candidate verbs based on their syntactic behaviour (i.e., participation in diathesis alternations and subcategorisation properties) and semantic characteristics.

First, for each English member verb of a given class, its predominant sense (i.e., the most frequent sense in WordNet, following Sun et. al. (2008)) was translated^2^, provided it fitted in semantically with the rest of the class (otherwise, the next adequate sense listed in WordNet was chosen instead). If several translations were identified for one English verb, each of them was considered and listed as a candidate for selection. To identify all relevant candidates, participants could consult the WordNets available in the target language. To make up for verbs which could not be translated, close synonyms of already identified candidate verbs could be added.

**Table 1:** Participant questionnaire, each question asked per VerbNet class

Q1	How many English verbs can be directly translated?
Q2	How many candidate verbs have been derived from external sources, not direct translation?
Q3	How many candidate verbs in the target language have been identified (pre-selection)?
Q4	What proportion of VerbNet frames can be translated into the target language?
Q5	How many additional frames have been identified for the target language?
Q6	How many candidate verbs have been selected based on frames?
Q7	How much time did you spend on the task [mins]?

In the selection stage of the process, in order to determine the membership criteria for each class, the first step involved translation of syntactic frames and diathesis alternations listed in VerbNet for each of the gold standard classes from English into the target language, taking into account the semantic roles and selectional restrictions specified for each VerbNet frame. Frames and diathesis alternations which could not be translated or were not applicable to the target language were recorded, and the number of translatable frames for each class was reported. Next, all other subcategorisation frames and diathesis alternations (not listed in VerbNet) possible for the candidate verbs were considered, keeping in mind Levin’s (1993) criterion that diathesis alternations result in the same (or extended) sense of the verb. Valency dictionaries could be consulted for this purpose, where available. The frames and diathesis alternations in which most verbs could take part were chosen as membership criteria for the class in question, and the candidates which could not appear in them were eliminated.

By using the original VerbNet frames as the starting point for evaluation of class membership in the target language, the selection process was systematised, with each translator considering the same syntactic and semantic criteria regardless of their linguistic expertise or target language. Although the basic procedure is language-independent, the method allows for certain language-specific adjustments and additional constraints on class membership criteria, defined in the selection phase of the task. For instance, in languages where morphological case is prominent (e.g., Polish or Croatian), the original VerbNet frames are further specified for the case marked on the verb’s argument. Whereas for languages with a word order distinct from that found in English (like the SOV word order in Japanese), the original VerbNet frames would be modified to match the default word order in the target language.

### Test case: Polish

Polish, a West Slavic language closely related to Czech and Slovak, was chosen as test language in order to probe the limits of the method when applied to languages typologically distant from VerbNet’s original language. Some of the main linguistic characteristics which distinguish it from English are verbal aspect and its extensive case system, which have been argued to pose a challenge to Levin-style classification (Gawronska 2001; Pala and Horák 2008).

**Table 2:** Average counts per each translated VerbNet class for linguist and non-linguist participants in the Polish task

Question	Q1		Q2	Q3	Q4		Q5	Q6	Q7
	Mean	$\upsigma$	Mean	Mean	Mean (%)	$\upsigma$	Mean	Mean	Mean
Linguists	11.55	0.24	3.92	14.80	71.4	0.34	0.90	11.08	23.0
Non-linguists	11.39	0.51	4.35	17.37	75.8	0.64	0.02	11.45	40.6
Overall	11.47	0.38	4.14	16.09	73.6	0.50	0.46	11.27	31.9

### Data analysis

Average counts for the Polish data are reported in Table 2. A high proportion of English verbs could be directly translated, with the average of 95.6% and a slightly higher translatability reported by linguists than non-linguists. This is a promising result suggesting that if translation was automated, only a small proportion of original member verbs would be lost. Approximately 4 synonyms were derived from external sources (e.g., Polish WordNet) for each class, which suggests that WordNet synonyms can be used to make up for verbs not translated directly. Approximately 16 candidate verbs were listed for each class in the first stage of the task, ranging from 7 to 35, with non-linguists providing on average more translation equivalents than linguists.

A significant proportion ($\approx$ 74%, and 100% for classes REMOVE, SEND and PEER) of VerbNet frames could be directly translated into Polish, and a slightly higher translatability was reported by non-linguists. Although certain obstacles to direct frame transfer remain, this is an encouraging result suggesting that a significant proportion of syntactic frames and diathesis alternations are shared by the two languages, and therefore the English VerbNet could be used to facilitate the creation of a similar verb lexicon in Polish. In fact, on average scarcely any additional Polish-specific frames were used as selectional criteria across participants, which suggests that those derived from VerbNet (with necessary Polish-specific adjustments) were sufficient as evaluation criteria of class membership. It should be noted, however, that the fact that the participants without linguistics training did not use additional language-specific frames may be a consequence of their lack of expertise, which helped those with linguistic background to identify Polish-specific characteristics of verb classes in question.

Average standard deviation across 17 classes was calculated for linguists and non-linguists^3^ to examine the variation in translatability judgments for member verbs (Q1) and VerbNet frames (Q4). As other questions were open-ended, with no limits imposed, variation was expected and is not considered indicative of consistency of participants’ judgments. For both verbs and frames linguists were more consistent in their translatability judgments, while larger variation was found in non-linguists. All 17 classes were successfully translated into Polish and on average 30% of candidates were discarded based on non-participation in syntactic frames and diathesis alternations. The average number of verbs per class in the final Polish classifications across participants is 11.3. The average time dedicated to the translation and selection of one class is $\approx$ 32 min, however, it was substantially shorter for linguists (23 min) than non-linguists (41 min). Linguistics training was found to considerably facilitate and speed up the translation and selection process, even if its lack does not make its completion impossible. Overall, the average time necessary to complete the whole task is 9 hours.

**Table 3:** Pairwise agreements between 6 participants for Polish (1: pilot, 2–3: linguists, 4–6: non-linguists) using: (i) percentage agreement (Per), (ii) Cohen’s kappa (Coh), and (iii)LSpa. Average pairwise Per is 71.6%, Coh 0.404, and average pairwise LSpa is 63.2%

	1&6	1&5	1&4	1&3	1&2	2&6	2&5	2&4	2&3	3&6	3&5	3&4	4&6	4&5	5&6
Per (%)	70.4	64.3	63.7	87.9	76.1	71.4	67.2	67.8	83.8	72.3	66.2	68.2	69.1	79.0	67.2
Coh	0.351	0.217	0.207	0.741	0.512	0.419	0.335	0.348	0.672	0.422	0.294	0.336	0.347	0.555	0.304
LSpa (%)	63.3	58.5	58.9	83.4	67.0	59.4	57.0	57.5	74.2	62.7	57.3	59.7	59.7	72.5	57.5

### Inter-annotator agreement

In order to examine the consistency among the translators, several measures of inter-annotator agreement (IAA) were calculated^4^: the average pairwise percentage agreement, the average pairwise Cohen’s kappa (Cohen 1960; Carletta 1996), Fleiss’ kappa (Fleiss 1971), and Krippendorff’s alpha (Krippendorff 1980). In order to apply these measures to the data, a pool of all (314) verbs listed in the final versions of classifications by the 6 translators was created. For every class, each verb listed by at least one person was assigned to one of the two mutually exclusive categories, ‘selected’ or ‘discarded’, for each of the 6 translators, numbered 1–6, with 1 assigned to the pilot translation, 2–3 to the linguist participants and 4–6 to the non-linguist participants. The average percentage agreement (i.e., the percentage of cases on which the translators agree) obtained is 71.6%. The individual pairwise agreement scores are shown in Table 3. Overall, the linguists’ choices aligned more with the pilot translation than those of non-linguists, and had high degree of overlap between themselves ($\approx$ 83.8%). However, high agreement is also reported between two of the non-linguist participants (79% between participant 4 and 5). Since percentage agreement does not take into account the agreements obtained by chance, measures which correct for random agreement were also calculated. The average pairwise Cohen’s kappa and Fleiss’ kappa values are both 0.404, which can be interpreted as borderline moderate agreement (Landis and Koch 1977) or fair agreement (Fleiss 1971). Krippendorff’s alpha was 0.405 which is naturally low due to the open-ended nature of our task: the participants could provide any number of translation equivalents for each English verb and there was no limit imposed on the number of synonyms which could be added. Moreover, no fixed class size was imposed for the final version of the gold standard. This led to varying numbers of candidate verbs considered and selected, which had a big impact on the agreement between translators. If, for instance, one Polish candidate verb was considered and selected by only two translators, it was automatically assigned the category ‘discarded’ for all other translators. As a consequence, providing more synonyms (especially those less common) for a given verb, although beneficial for the comprehensiveness of the ultimate gold standard, was likely to negatively affect the agreement between translators.^5^ This is why the relatively low IAA score obtained should be interpreted with caution, bearing in mind the open-ended nature and difficulty of the task.

**Table 4:** Average pairwise LSpa IAA scores per each VerbNet class in Polish translation experiments

9.1-PUT	13.5.1 GET	22.2-AMALGAMATE	31.1-AMUSE	37.3-MANNER OF SPEAKING
58.3%	65.0%	55.9%	62.8%	59.4%
10.1-REMOVE	18.1-HIT	29.2-CHARACTERIZE	37.7-SAY	40.2-NONVERBAL EXPRESSION
60.7%	52.1%	51.5%	43.3%	76.3%
11.1-SEND	30.3-PEER	36.1-CORRESPOND	51.3.2-RUN	45.4-CHANGE OF STATE
64.9%	81.0%	72.3%	82.1%	68.3%
43.1-LIGHT EMISSION		47.3-MODES OF BEING (+MOTION)		
69.0%		52.5%		

In light of this issue, another agreement measure (labelled LSpa here) was computed which accommodates the unrestricted nature of verb translation. The metric was introduced by McCarthy and Navigli (2007) and used for evaluation of agreement between annotators in an English lexical substitution task and its cross-lingual variant (Mihalcea et al. 2010). The setting is similar to the one presented here: five English native-speaker annotators had to identify an alternative substitute word or a phrase for a target word in a certain context and any number of substitutes (up to three) could be provided. Pairwise agreement between each pair of sets of substitutes for each item ($p_1, p_2 \in P$) from each possible pairing (*P*) is computed as $=\frac{\sum _{p_1,p_2 \in P}\frac{p_1 \cap p_2}{p_1 \cup p_2}}{|P|}$.

In order to apply this metric to our Polish data, each English class of 12 verbs was treated as one item and the Polish member verbs listed by each participant constituted sets of substitutes. Thus, pairwise agreement between the verbs selected for each class of the Polish resource could be calculated for each pairing of translators. The results are promising: the total agreement for all 255 possible pairs of sets (17 classes, 15 possible pairings of translators) is 63.2%. This exceeds that reported by McCarthy and Navigli (2007) for the LexSub task (27.75%), as well as that obtained by Kremer et al. (2014) (19.3 and 24.6% for a smaller subset of data) and 27.8% on a cross-lingual variant of the task (Mihalcea et al. 2010). Lexical substitution tasks are different from ours. In our experiment the participants were instructed to provide translation equivalents as close to the particular WordNet sense in question as possible, while in lexical substitution tasks any word which could replace the target word in a given context without changing the meaning of the sentence is a valid substitute, allowing for more flexibility and hence, more variation. Nevertheless, the substantial agreement in our free response task suggests that the guidelines are coherent enough to yield consistent classifications. The pairwise LSpa scores shown in Table 3 confirm the observations from simple percentage agreement, with more overlap between linguist participants and the pilot translation and between themselves compared to non-linguists. The results obtained for class-based average pairwise LSpa agreement (Table 4) show most overlap in the classes RUN and PEER and the least in classes SAY and CHARACTERISE.

In order to produce the final version of the Polish resource, the candidate verbs selected by all participants were assessed against the criteria defined for a given class in the pilot classification, as most expertise was put into its creation. The final resource includes 258 verbs in 17 classes, with class size ranging from 7 to 21 members (15.2 on average).

### Data interpretation

Two main sources of difficulties for the translation process have been identified. Firstly, certain features of Polish grammar pose a challenge for the direct transfer of classes and necessitate language-specific considerations. For instance, VerbNet frames including prepositional phrases (5) had to be split further into several syntactic contexts specified for the case marked on the verb’s complement (6), as in the case of PUT class:

**Table:** 

5)	I put the book on/under/near the table.	6)	a)	Położyłam książkę na stole.		
	Agent V Theme (+LOC) Destination			put	book	on table.LOC
	NP V NP PP.DESTINATION		b)	Położyłam książkę pod stołem.		
				put	book	under table.INS
			c)	Położyłam książkę obok stołu.		
				put	book	near table.GEN

As there is no fixed relationship between Polish prepositions and the case marked on the complement they govern, the case required will vary from one context to another depending on the verb present and the intended meaning of the predicate. This is an important complication for the frame translation process, as Polish requires more fine-grained distinctions and more detailed descriptions for frames than ‘NP V NP PP.DESTINATION’ found in VerbNet, which was noted by all participants and would have to be taken into account in construction of a comprehensive resource.

Moreover, the differences between the diathesis alternation inventories in both languages resulted in a lower translatability of frames in certain classes (e.g., HIT (54%), RUN (39%), CORRESPOND (55%)). For example, the *with/against* alternation, characteristic of the English HIT verbs (7), can only be expressed in Polish by means of case marking on the Instrument (8):

**Table:** 

7)	a)	Maria hit the stick against/on the rock.	8)	a)	Maria uderzyła kijem o kamień.
		Agent V Instrument against Patient			Agent V Instrument.INS o Patient
		NP V NP PP			NP V NP.INS PP
	b)	Maria hit the rock with the stick.		b)	Maria uderzyła w drzewo kijem.
		Agent V Patient with Instrument			Agent V w Patient Instrument.INS
		NP V NP PP			NP V PP NP.INS

Whereas the conative (9) and resultative^6^ (11) alternations do not exist in Polish at all, and a similar meaning can only be conveyed using imperfective aspect (10) or an instrumental NP (12).

**Table:** 

9)	a)	Maria hit the tree (with the stick).	10)	a)	Maria uderzyła w drzewo (kijem).
		Agent V Patient (with Instrument)			Agent V w Patient (Instrument.INS)
		NP V NP (PP)			NP V(perfective) PP (NP.INS)
	b)	Maria hit at the tree (with the stick).		b)	Maria uderzała w drzewo (kijem).
		Agent V at Patient (with Instrument)			Agent V w Patient (Instrument.INS)
		NP V PP (PP)			NP V(imperfective) PP (NP.INS)

**Table:** 

11)	Maria kicked the gate open.	12)	Maria otworzyła bramę kopnięciem.
	Agent V Patient Result		(‘Maria opened the gate with a kick’)
	NP V NP ADJP		Agent V Patient Instrument.INS
			NP V NP NP.INS

Secondly, varying levels of linguistic expertise and English language proficiency in the participants made the task more challenging for some translators and resulted in discrepancies in the final classes. For example, mismatches in translatability judgments were noticeable in the case of the middle construction, which some participants erroneously translated into an impersonal construction instead. Overall, participants with linguistics training tended to be more sensitive to the nuances of meaning expressed by different diathesis alternations. In several cases loose paraphrases of the original frames were accepted as direct adaptations of given constructions, which produced inflated translatability judgments. The highest inter-annotator agreement was found in classes RUN (82%) and PEER (81%), where the translation and selection task was made easier by well-defined semantics of the class in question and concrete meanings of verbs, which could be rendered by a single Polish translation equivalent (e.g., ‘run’—*biegać*, ‘swim’—*pływać*, ‘listen’—*słuchać*, ‘stare’—*gapić siȩ*), thus leading to a high overlap between the candidates identified. What is more, the same alternations were selected as class membership criteria by all participants (e.g., for the PEER class verbs, ‘NP V’ and ‘NP V PP (na/w+ACC)’), and the same decisions regarding verbs’ participation in these frames were made. In more vaguely characterised classes (e.g., CHARACTERISE), deciding on an adequate translation equivalent was subject to more variation, which resulted in greater mismatches between class members selected (51% agreement). As linguistic expertise proved helpful in the selection phase and was found to significantly speed up the task, in the next stage of the experiment where the methodology is applied to other languages, the task was performed by translators with linguistic background.

## Multilingual transfer of verb classes

Based on the Polish experiment it was concluded that the rationale behind VerbNet classes, according to which verbs are grouped based on their participation in syntactic frames and diathesis alternations, as well as our translation method, allowing for certain language-specific adjustments, are indeed applicable to Polish and hence, possibly to other languages. The aim of the experiment was to assess that applicability by performing the translation task for a set of languages differing from English with respect to certain features relevant to verb classification: Croatian, Italian, Mandarin, Japanese, and Finnish. The procedure followed in the Polish experiment was replicated, however, with one native-speaker translator (with some linguistic background) performing the task for each language, following the translation guidelines. WordNets and valency dictionaries, where available, could be used to identify additional synonyms and language-specific frames.

**Table 5:** Results and statistics of gold standard translation to five other languages

	Croatian	Mandarin	Japanese	Italian	Finnish
Percentage of translated English verbs (Q1)	87%	98%	82%	96%	92%
Number of synonyms extracted from external resources (per class) (Q2)	4	0	5	2	1.3
Number of candidates identified prior to selection (per class) (Q3)	16.4	14.5	8.2	12.2	16.2
Percentage of translated VerbNet frames (Q4)	87%	97%	78%	83%	71%
Total number of language-specific frames identified (Q5)	11	63	0	0	3
Number of translated classes (Q5)	17	17	17	17	17
Percentage of candidates discarded based on frames (Q6)	2%	2%	7%	14%	28%
Total number of verbs in the final gold standard (Q6)	274	241	129	177	199
Average class size in the final gold standard (Q6)	16	14.2	7.6	10.4	11.7

### Results

The results obtained for Croatian, Italian, Mandarin, Japanese, and Finnish reveal a significant potential for cross-lingual transfer of English verb classes, as evident from Table 5. Across the languages studied, all 17 classes were successfully transferred with a high proportion of member verbs and frames directly translated into the target language. For all languages apart from Mandarin external sources proved useful to derive additional synonyms of member verbs and make up for those English verbs which could not be directly translated. As in the Polish experiment, certain linguistic properties had to be taken into account during the translation of VerbNet frames; however, they had a smaller impact on the ultimate verb selection process, as frames and diathesis alternations specific to the language in question could be used as evaluation criteria for a given class. According to the feedback provided by the participants, identifying alternations in their native language and using them for evaluation of candidate verbs was the most challenging part of the task. As seen in Table 5, no language-specific frames were recorded for Japanese and Italian, and only three were taken into consideration in the case of Finnish. This may have been due to the translators’ bias towards English: using the original English VerbNet frames as the starting point for frame translation and identification was likely to make the task of distinguishing language-specific phenomena and characteristics more difficult. The lack of additional selection criteria affected the size of certain classes: in the case of HIT, AMALGAMATE, and CORRESPOND, only 2 or 3 verbs were listed in the final Finnish classes, and the rest of the candidates were discarded based on their non-participation in the VerbNet frames translated from English. Identifying Finnish-specific syntactic criteria for class membership in these cases would have helped create more comprehensive groupings of verbs in the final classification. This is why this stage in the process could especially benefit from both linguistic expertise and resources (e.g., valency dictionaries) specific to the language in question, and their lack was noted as a source of difficulty. Particularly in Croatian and Mandarin the unavailability of high quality resources to aid the task of identifying synonyms and syntactic frames was noted as an obstacle. The final versions of classifications range in size from 274 verbs in Croatian to 129 verbs in Japanese, with average class size ranging from 16 to 7.6. The differences in the number of verbs in the final classes result from the differences in verb inventories available in each language, rather than translatability of the English gold standard. For example, while 82% of English class members were successfully translated into Japanese, many of the finer distinctions made by English verbs are lacking in Japanese, where the same verbs convey the meaning of distinct English words: for example, although 10 English LIGHT EMISSION verbs were directly translated, only 5 distinct Japanese candidates were listed (*kagayaku, hirameku, moeru, kirameku, hikaru*).

**Fig. 1:**
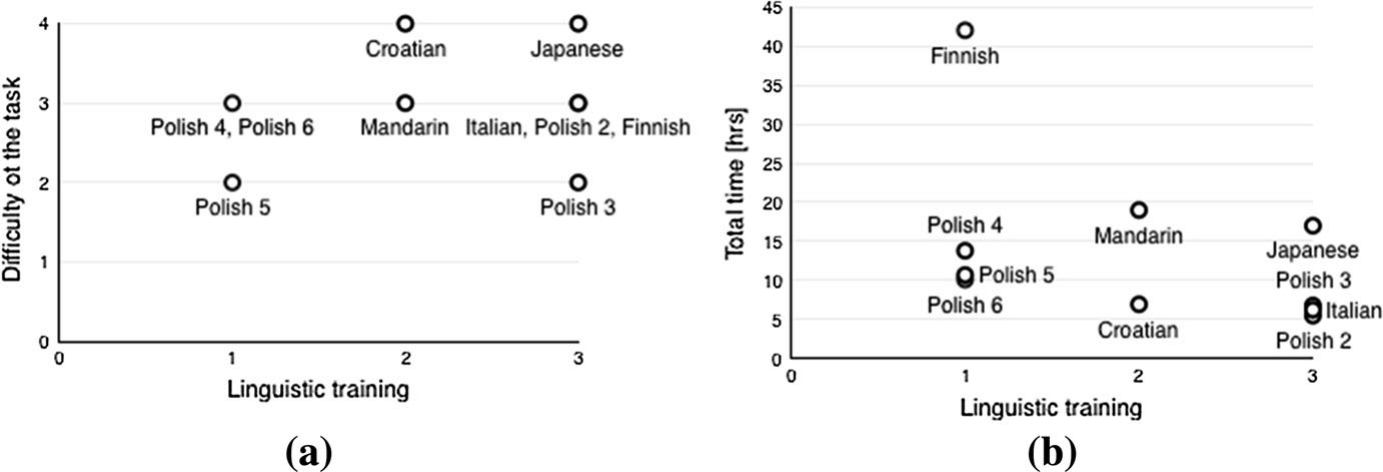
**a** Difficulty and **b** total time spent on the task for different annotators (Q7)

In the feedback survey the participants in the Polish and multi-lingual experiment were asked to rate the difficulty of the task on the scale 1–4 (1: *easy*, 2: *moderate*, 3: *difficult*, 4: *very difficult*) and note whether they had linguistics training (1: *no*, 2: *some* (informal or non-university level), 3: *yes* (i.e., formal university-level training)). Most participants judged the task as difficult regardless of linguistics training (Fig. 1). The task proved most difficult for the Croatian and Japanese translator, while two Polish participants, with and without linguistic background, found the task moderately challenging. Generally, linguistic training contributed to shortening the time required for the completion of the task (Fig. 1), with the three shortest times reported for two Polish linguists and the Italian translator, with considerably longer times recorded by Polish non-linguists. However, Finnish stands out as the most time-consuming, despite the translator’s linguistic training: the total duration of the task (42h) is more than double the time needed to complete the process for the second most time-consuming language, Mandarin (19h). The process was considerably faster for Croatian (7h) and Italian (6h 15min).

### Comparison of manual versus automatic approach

The presented translation method is aimed at obtaining high-quality gold standard classes across a range of typologically diverse languages by leveraging an existing English resource to speed up the process, compared to the time required for manual development of such resources from scratch (e.g., several years in the case of English VerbNet). The hypothesis behind this approach was that performing the task manually would guarantee obtaining accurate enough gold standard classes that could be used in future experiments, for example, for evaluation of automatic verb clustering tasks. In order to verify this assumption, we wanted to compare the results obtained using the presented method with those potentially obtainable (semi-)automatically, for example, using mappings between WordNet senses and VerbNet from the Predicate Matrix (Lacalle et al. 2016). In order to compare the two approaches and evaluate this alternative method, we chose Mandarin as the test language and used the Predicate Matrix in order to obtain candidate verbs for all of the 17 English classes used in the study. Starting from pairings of English verb (from the English gold standard) and VerbNet class, we looked up corresponding WordNet 3.0 synsets in the Predicate Matrix, and subsequently used the links between Princeton WordNet and the Chinese Open WordNet (Wang and Bond 2013) to obtain Mandarin candidate verbs. In order to evaluate these candidates, two native speakers of Mandarin performed a blind test in which they were presented with a shuffled list of Mandarin verbs obtained both through manual translation and via the Predicate Matrix and asked to mark those which they did not consider to be good equivalents of the English gold standard verbs.

**Table 6:** Comparison of the number of candidates and noise in classes obtained using the manual translation method and the Predicate Matrix (PM)

	Number of candidates		Number of noisy candidates		Number of overlapping candidates
	Manual	PM	Manual	PM	Manual & PM
10.1 REMOVE	13	32	2	9	4
13.5.1 GET	15	44	1	7	8
18.1 HIT	15	19	0	2	4
22.2 AMALGAMATE	14	39	0	5	8
30.3 PEER	14	42	0	5	8
36.1 CORRESPOND	16	28	1	1	6
40.2 NONVERBAL EXPRESSION	12	21	0	0	5
43.1 LIGHT EMISSION	12	35	3	5	10
47.3 MODES OF BEING +MOTION	15	24	0	3	5
9.1 PUT	13	32	0	4	7
11.1 SEND	16	33	0	2	11
29.2 CHARACTERIZE	7	18	0	2	1
31.1 AMUSE	14	37	0	0	7
37.3 MANNER OF SPEAKING	15	27	0	4	6
37.7 SAY	18	37	0	3	7
45.4 CHANGE OF STATE	15	20	0	3	2
51.3.2 RUN	14	62	0	9	11
Total	238	550	7	64	110
% of all manual$^{\mathrm{a}}$ or PM$^{\mathrm{b}}$ candidates			${2.9\%^{\mathrm{a}}}$	${11.6\%^{\mathrm{b}}}$	${46.2\%^{\mathrm{a}}}$

^a^Percentage of all manual candidates

^b^ Percentage of all Predicate Matrix (PM) candidates (as stated in the bottom-left corner of Table 6; the letters link the percentage values in the bottom row to words ‘manual’ and ‘PM’, respectively, in the description in column 1)

The percent agreement between the two native-speaker evaluators was 92% (with a kappa coefficient of 0.84). The results of this evaluation (Table 6) showed that $\approx$ 12% of the Mandarin candidate verbs obtained via the Predicate Matrix were judged as noisy, and $\approx$ 3% were judged as such in the output of manual translation. At the same time, the automatic method using the Predicate Matrix picked up 46% of the candidate verbs manually identified by the Mandarin translator using the method presented in this study. This suggests that the Predicate Matrix can be useful to generate additional candidates; however, the output of the automatic method is noisy and, what is important, it misses out over half of the gold standard candidates identified manually using the translation method. The poorer accuracy of the automatically generated classes with respect to those obtained via our translation method makes their utility as an evaluation resource in clustering experiments much lower. Another limitation of the automatic method is that access to a WordNet in the target language is necessary. These are still unavailable for the majority of languages, which is why manual translation has the advantage of being potentially universally applicable, regardless of the resources available. Nevertheless, the Predicate Matrix can serve as a useful auxiliary tool. According to the feedback provided by the evaluators, the verbs identified by both methods (i.e., the overlapping cases) were particularly good candidates for each class. The Predicate Matrix could therefore be used to identify prototypical class members within the manually obtained sets of translations that would carry more weight in machine evaluation.

### Discussion of the impact of language differences

The languages considered in this study were sampled across language families in order to diversify the typological properties tackled and provide a probe into the cross-lingual applicability of the translation method and guidelines. In this section we look at some of the issues raised by these languages in terms of different morphosyntactic alignments, degree of morphological complexity, or lexicalization patterns, among others, each of them relevant to certain stages of the verb class translation task.

#### Morphosyntactic alignments and morphological complexity

As a Slavic language with rich morphology and a flexible word order, Croatian poses similar challenges to verb class translation as Polish. For instance, frames including PPs like NP V NP PP.DESTINATION (*I put the book on/under/near the table*), require distinguishing between the different case marked on the NP depending on the preposition used, whereas diathesis alternations including an instrumental PP in English (NP V NP PP.INSTRUMENT, *Paula hit the ball with a stick*) had to be modified to NP V NP NP.INSTRUMENT (*Paula je udarila loptu štapom*) with the preposition dropped and an NP marked with instrumental case. In Mandarin, a Sino-Tibetan SVO language, special attention is required by serial verb constructions, where two or more verbs (or VPs) appear concatenated together, often involving the so-called coverbs, sharing properties of verbs and prepositions, and thus posing a challenge for classification. Japanese SOV word order, with verbs constrained to the clause-final position and particles indicating the grammatical function of words in the clause, requires adjustments during the translation of the English SVO frames. Whereas Finnish, an agglutinative nominative-accusative language that belongs to the Uralic family and is characterised by an extensive case system and a flexible word order, makes use of suffixes to express grammatical relations and employs different verb forms to convey transitivity distinctions (e.g., *kuivata* (‘to dry’, transitive) and *kuivua* (‘to dry (out)’, intransitive), which requires language-specific treatment for the purposes of classification. Finally Italian, characterised by the same basic word order as English but allowing for much more flexibility, differs from English in its diathesis alternations inventory, which resulted in a lower translatability of VerbNet frames: for instance, it lacks the resultative construction (*I kicked the door open*) and the dative/benefactive alternation (*I bought a dress for Mary—I bought Mary a dress*). Moreover, fewer Italian candidates were identified on average for each class than in Croatian or Mandarin, mostly due to the fact that distinct English verbs translated into the same Italian verbs or into phrases and idiomatic expressions rather than single words, which resulted in a smaller average class size in the final gold standard.

#### Lexicalization patterns and varying verb inventories

While different morphosyntactic alignments may affect direct translatability of diathesis alternations, they are less of a complication for the translation of member verbs. Cross-linguistic differences which seem particularly relevant to the transfer of verb classes through translation are found in lexicalization patterns (i.e., regularities in the way conceptual components are encoded in lexical items), in particular, the differences in what elements of an event are encoded in or outside the verb. The assumption which underlies typological study of these patterns is that languages analyse similar events using similar types of conceptual components and that cross-linguistic variation is constrained, it is therefore possible to classify languages into types based on the lexicalization options which they permit (Levin and Hovav 2015). Talmy’s (1985) examination of directed motion verbs reveals that languages tend to encode the semantic elements of ‘Path’ and ‘Manner’ in one of two ways: either conflating ‘Manner’ with ‘Motion’ inside the verb, as is typical of English (*The ball rolled down the slope*), or expressing ‘Motion’ together with ‘Path’ in the verbal root, which is characteristic of Romance languages, where ‘Manner’ may be expressed as a gerundive adjunct (13) (Folli and Ramchand 2005). What is important, in languages displaying the latter pattern, descriptions of directed motion events where ‘Manner’ is lexicalized in the verb are not permitted (Carter et al. 1988; Levin and Rapoport 1988). The PP in (14) (Folli and Ramchand 2005) can only be understood as the location, while in the English translation it can be interpreted as either the location or the goal of motion:

**Table:** 

13)	La	botte	è entrata	nella	cantina	rotolando	14)	La	barca	galleggiò	sotto	il	ponte
	the	barrel	entered	in the	basement	rolling		the	boat	floated	under	the	bridge
	‘The barrel rolled into the basement’							‘The boat floated under the bridge’					

An important consequence of these diverging lexicalization patterns are the differences in verb inventories: languages presenting the first pattern tend to have large repertoires of verbs expressing motion occurring in various manners, while those with a preference for the second pattern will have more verbs describing motion along a certain path (Talmy 1985). This is of direct relevance to verb class transfer, as classes such as MODES OF BEING WITH MOTION or RUN, including an array of English ‘manner’ motion verbs, may be less applicable to languages where manner is expressed outside the verb. The pattern characteristic of Italian is also found in Japanese, where ‘manner’ is expressed in a participle and ‘path’ is lexicalized in the verb:

**Table:** 

15)	Taro wa	kawa o	aruite watat-ta
	Taro TOP river ACC walk cross-PST		
	‘Taro walked across the river’ (Matsumoto 2003)		

According to Wienold (1995), Japanese is one of the purest ‘path’ languages, with only 13 monomorphemic manner-of-motion verbs (Matsumoto 1997), in contrast to Germanic languages: Levin (1993) records over 100 English manner-of-motion verbs, and a similarly high number can be found in German (Snell-Hornby 1983). While such basic manner-of-motion verbs as ‘run’ or ‘fly’ can be found in most languages (Wienold 1995), ‘manner’ languages will tend to make much subtler distinctions regarding the way in which an action is performed. The gold standard translated in the experiment included only a selection of verbs from each Levin class, and enough translation equivalents could be provided both in Italian and Japanese. If creation of a comprehensive classification of all verbs in the target language was the goal, grouping ‘Path’ verbs together in languages displaying such lexicalization pattern might be more appropriate. The distinction in preferences for ‘Path’ or ‘Manner’ constructions found across languages can explain why translation of English motion verbs will be easier into some languages than others. An analogous pattern as in English is found in Polish, Croatian (according to Talmy (1985), in all Indo-European languages excluding Romance) as well as Mandarin and Finnish, which tend to encode ‘Path’ in the so-called ‘satellites’ (i.e., constituents in a sister relation to the verb root other than NP or PP complements (Talmy 2000)), e.g., English particles like *run* *out*, Mandarin directional verbal complements like *piào* *guò* (*float past*), as opposed to the languages in which ‘Path’ is encoded by the verb (Matsumoto 2003). The examples in Mandarin (16), Polish (17, ‘Path’ is encoded by the prefix *w-*) and Finnish (18, where ‘Path’ is expressed by the case marker suffix *–sta*) illustrate ‘Manner’ and ‘Motion’ lexicalized inside the verb, with ‘Path’ expressed outside:

**Table:** 

16)	Wǒ	yòng	zuó jiǎo	bǎ	qiú	tī	guò	le	cāo-chǎng
	I	use(-ing)	left foot	OBJ	ball	kick	across	PERF	field
	‘I kicked the ball across the field with my left foot’

**Table:** 

17)	Barman	w-toczył	beczkȩ	do	piwnicy.
	bartender	in-rolled	barrel	into	basement
	‘The bartender rolled the barrel into the basement.’

**Table:** 

18)	Puunkolo-sta	lehahti	pöllö
	tree hole-ELA	whip.IMP.3.SING	owl.NOM
	‘An owl whipped from the tree hole’ (Pasanen , Pakkala-Weckstöm 2008)		

Languages with a preference for lexicalizing manner of motion are often found to have an extensive inventory of verbs expressing manner in general, as can be seen in Levin classes MANNER OF SPEAKING or PEER. This is reflected in the especially numerous MANNER OF SPEAKING class in Finnish (24 verbs) and MODES OF BEING WITH MOTION class in Croatian (22 verbs). In contrast, for a ‘path’ language Japanese, only 6 PEER verbs and 5 MANNER OF SPEAKING verbs were identified. Similarly, 7 PEER verbs were listed in Italian, again suggesting a more restricted inventory of manner verbs. Languages such as Italian and Japanese tend to make subtle meaning distinctions like those found in English manner verbs in adverbials; for example, in onomatopoeic terms related to manners of motion, e.g., Japanese adverbs *choko-choko* (‘with sharp rapid movements’), *pyon-pyon* (‘with repeated hops’) (Matsumoto 2003).

#### Causativity and transitivity

Further differences arise in ‘change of state’ verbs: while English verbs such as *dry*, *melt* and *open* can appear both in non-causative and causative contexts (i.e., the causative-inchoative alternation^7^, e.g., *He opened the door/The door opened*), Croatian, Polish (19), and Italian (20) require a reflexive pronoun to accompany the non-causative verb. The basic verb form in Italian, Polish and Croatian is agentive (transitive), whereas Japanese verbs referring to states are mainly lexicalized in the non-causative type and a causative verb form, with an inflection added to the stem, is required to express the agentive (21b) (Talmy 1985). In Finnish, however, two different verbs express the transitive (22a) and intransitive meaning (22b).

**Table:** 

19)	a)	Słońce	stopiło	śnieg.	20)	a)	Aprì	la	porta.
		sun.NOM	melted	snow.ACC			opened	the	door
		‘The sun melted the snow.’					‘He opened the door.’		
	b)	Śnieg	stopił	siȩ.		b)	La porta	si	aprì.
		snow.NOM	melted	REFL			the door	REFL	opened
		‘The snow melted.’					‘The door opened.’

**Table:** 

21)	a)	Doa	ga	ai-ta			22)	a)	Bill	kuivasi	vaatteet.
		door	SUBJ	open-PST					Bill	dried	clothes
		‘The door opened’							‘Bill dried the clothes.’		
	b)	Kare	wa	doa	o	ak-e-ta		b)	Vaateet	kuivuivat.	
		he	TOP	door	OBJ	open-TR-PST			clothes	dried	
		‘He opened the door’							‘The clothes dried.’		

The differences in the way semantic elements are encoded in surface form are reflected in the verb inventories available in the languages in question and the syntactic constructions in which they can appear. For example, in Polish (and several other Slavic languages), the reflexive inchoative verbs^8^ (19b) form equipollent pairs with non-reflexive variants (‘to melt’: *stopić się—stopnieć*), which can only appear in intransitive contexts:

**Table:** 

23)	a)	*Słońce	stopniało	śnieg.
		sun.NOM melted		snow.ACC
	b)	Śnieg	stopniał.	
		snow.NOM melted		
		‘The snow melted.’		

Although the so-called double inchoatives (i.e., the reflexive and non-reflexive variants of an inchoative verb) share meaning, they differ in their syntactic behaviour: only the reflexivising verbs can participate in both the intransitive and the transitive construction, which sets them apart from the synonymous non-reflexive variants based on Levin’s (1993) criterion, i.e., participation in the same alternations. This finds confirmation in the Polish and Croatian classifications: in both languages the participants used the causative/inchoative alternation as a membership criterion for the CHANGE OF STATE class and listed the transitive reflexivising variants as members of the final class, eliminating their non-reflexive intransitive counterparts. Analogously, in Finnish the transitive verbs were listed as class members, while the intransitive candidates were discarded.

Similar considerations are required in the case of Finnish causative verbs. Derived by attaching a causative suffix *–(U)ttA-* to verbal (or nominal) stems (e.g., ‘to jump’, *hypätä*—‘to make someone/something jump’, *hyppäyttää*), the so-called curative causatives take two active arguments and encode the meaning of ‘x makes y do something’ (24b):

**Table:** 

24)	a)	NP V PP.location				b)	NP V NP PP.location				
		Hevonen	hyppäsi	aidan	yli		Tom	hyppäytti	hevosen	aidan	yli
		Horse	jumped	fence	over		Tom	jumped	horse	fence	over
		‘The horse jumped over the fence’					‘Tom jumped the horse over the fence’				

In the Finnish experiment, the causative verb candidates for the RUN class identified prior to selection (*hyppäyttää, juoksuttaa, marssittaa, nelisyttää, liuuttaa, lennättää, uittaa*) had to be eliminated from the final class based on their participation in different syntactic frames than their basic intransitive forms listed as class members. To capture both the semantic similarity and distinct syntactic behaviour of the two types of verbs—the causatives and the intransitive basic forms in Finnish, or the double inchoatives in Polish—a comprehensive VerbNet-style classification would have to break up the original RUN or CHANGE OF STATE classes into subclasses, distinguished by the participation/non-participation in the causative syntactic contexts.

#### Cross-linguistic commonalities

As illustrated by these language-specific examples, cross-linguistic variation with regard to the features discussed may pose a range of challenges for translation of verb classes. Languages where transitivity contrasts are expressed with distinct verb forms with shared semantics but distinct syntactic behaviour or those exhibiting different lexicalization patterns will require a different architecture of classes and subclasses to capture the semantic properties encoded in the verb root. Moreover, languages with extensive case marking will require more fine-grained distinctions between syntactic contexts used as membership criteria for a given class.

Nonetheless, while language-specific adjustments are inevitable in the translation of syntactic frames, the high overall percentage of frames successfully translated in all languages suggests most of the them are shared and cross-linguistically valid. Notably, in all the languages considered all of the VerbNet frames listed for classes REMOVE and SEND were judged as translatable and applicable in the target language. For example, all languages studied allow ‘remove’ verbs to appear in frames such as NP V NP PP.SOURCE (*I removed the stains from the tablecloth*), as well as ‘send’ verbs to take prepositional phrases expressing ‘initial location’ and ‘destination’ (e.g., NP V NP PP.DESTINATION *I sent the parcel to New York*, NP V NP PP.INITIAL_LOCATION *I sent the parcel from Paris*). More shared frames are found outside these two classes, for instance, all the languages considered allow ‘put’ verbs to alternate between a ‘destination’ complement expressed as a prepositional phrase (NP V NP PP.DESTINATION *I put the plate on the table*) and as an adverb (NP V NP ADVP *I put the plate here/there*). The languages studied in the experiment constitute a small sample and therefore are not representative of all cross-linguistic variation as far as verb argument structure is concerned. However, by performing systematic analyses such as ours we may gain new insights into the concept of universality of verb frames and identify structures which are potentially cross-linguistically valid.

Typological study of lexicalization patterns can provide further insight into which aspects of the verb system in question may require language-specific treatment and where we may expect a high degree of translatability. For instance, it would be interesting to see how English verbs translate into languages which fall into Talmy’s third type, where ‘Motion’ and ‘Figure’ (i.e., the object undergoing movement) are lexicalized in the verb root, as it is the case in Navajo (Talmy 1985). In the present study, translation produced semantically and syntactically coherent classes for all languages examined. This supports the hypothesis about the cross-linguistic validity of the approach using diathesis alternations as diagnostics for shared syntactic and semantic properties of verbs. Further research is necessary to investigate the applicability of the method to some of the less-studied languages, including ergative and active-stative languages, whose realisations of the predicate-argument structure and lexicalization patterns diverge from those found in nominative-accusative languages such as English or Polish. By investigating the applicability of VerbNet-style classes to a typologically diverse set of languages and the commonalities between their verbal systems this work lays the foundations for further exploration of the universal set of verb frames and roles.

## Conclusion and future work

This study constitutes the first empirical investigation of the translatability of English VerbNet classes across typologically diverse languages. A systematic translation method was developed which is largely language-independent but allows for language-specific treatment of aspects of verb behaviour accommodated in the classification. Polish was chosen for the in-depth evaluation of the method aimed at achieving high accuracy of classification, which was subsequently applied to five other languages from different language families: Slavic (Croatian), Romance (Italian), Sino-Tibetan (Mandarin), Japonic (Japanese) and Uralic (Finnish).

The study was conducted in three stages. First, detailed, language-independent translation guidelines were developed and the task was completed by the pilot linguist. Next, five native speakers of Polish, two with linguistic training and three without it, completed the gold standard translation task following the guidelines and recorded information about every stage of the process, which provided quantifiable data for the assessment of the method. Finally, having motivated and tested the method on Polish verbs, another five participants performed the translation of verb classes into their native languages, Croatian, Mandarin, Italian, Japanese, and Finnish.

The pilot translation and the first experiment demonstrated that the rationale behind Levin-style classes and the translation method are applicable to Polish, however, certain language-specific adjustments have to be made in order to create a valid classification of Polish verbs. Although a high percentage (96%) of English member verbs could be directly translated, certain features of Polish grammar, such as aspect or reflexivity, need to be taken into account when selecting the appropriate translation equivalents. Moreover, due to the extensive case-marking on verbal arguments in Polish, syntactic frames and alternations derived from VerbNet need to be modified to capture the differences in case-marking on the argument NPs required by different verbs. The necessity of making such language-specific adjustments is what makes the process challenging for translators without linguistic background. As the translation and selection process required recognising fine distinctions between verbs based on subtle differences in syntactic behaviour and making subjective decisions about ambiguous cases, it would be very difficult to carry out the same task automatically.

Translators with linguistic training were more consistent in their choices than non-linguists and tended to be more sensitive to the nuances of meaning encoded in diathesis alternations. Kappa measures of agreement were impacted by the open-ended nature of the task. Application of McCarthy and Navigli’s (2007) metric to the per class choices from each participant resulted in a promising 63.2% agreement which compares favourably to lexical substitution tasks (McCarthy and Navigli 2007; Kremer et al. 2014; Mihalcea et al. 2010) and suggests that the guidelines produce reliable results from human translators and high-quality classifications.

The methodology for cross-lingual VerbNet class translation was further tested on five other languages: Mandarin, Japanese, Croatian, Italian, and Finnish. This selection investigated the applicability of the translation method to languages differing with regard to morphosyntactic alignment, case system or lexicalization patterns. Notably, for all languages a high percentage of English verbs could be directly translated, ranging from 82% in Japanese to 98% in Mandarin. A similarly high proportion of VerbNet frames was judged as translatable into the target language and the concept of diathesis alternations as evaluation criteria of verb class membership was found to be valid for all languages examined. Linguistic expertise was found to be helpful for the task, especially in the absence of high quality resources (e.g., valency dictionaries or WordNets). It was also found to support the finer-grained levels of the task (translation of frames and application of diathesis alternations) and inclusion of language-specific properties in the classification.

Across the languages studied, the results and feedback collected indicate the need for language-specific treatment of certain aspects of verb behaviour with regard to transitivity contrasts, word order or case marking in order to fully capture the semantic information and syntactic properties exhibited by verbs in the target language. However, at the same time, these idiosyncrasies can be accommodated in a Levin-style classification, as the gold standards produced demonstrate (Online Resource 2), thus supporting the hypothesis of its cross-linguistic applicability. The main advantage of the translation approach is that it allows creation of verb classifications in languages lacking NLP resources. However, in such cases language-specific expertise of the translator will likely be indispensable. Moreover, the Polish experiment showed that having several or even just a pair of translators perform the task could further benefit the task, producing a more comprehensive resource. Inter-annotator consultations would allow to complement the classification with additional synonyms and verify the decisions about acceptability of certain constructions, as well as help define adequate membership criteria for each class. While it is difficult to be sure that the open-ended nature of the task will account for every possible construction, our finding of reasonable agreement from linguists indicates that the classifications are reliable. It is possible that the resultant gold-standard classifications, envisaged for evaluation of automatic systems, could themselves be further populated by such systems. As illustrated by the results of the comparison of our translation method and automatic generation of candidates using the Predicate Matrix, the latter method can produce additional candidates, however, its output is noisy and requires post-hoc verification.

Our approach has certain limitations. As the method involves translatability and grammaticality judgments, it is to a significant extent subjective. What is more, as each translator is likely to use slightly different constructions as evaluation criteria, it is hard to systematise and may be prone to oversight. In order to constrain the evaluation and selection procedure, the method relies on using the original VerbNet frames (if translatable into the target language) as the primary evaluation criteria of class membership, guiding the process. However, as the next step involves language-specific considerations, these may differ from translator to translator; it is therefore beneficial to allow for consultations between them, likely to produce a more comprehensive and accurate resource.

The contributions of the presented research are of theoretical as well as practical nature. On the practical side, our translation methodology can be readily applied to build high quality VerbNets and/or gold standards for automatic verb classification in a more time-efficient way than when creating them from scratch. The guidelines and the classifications we have already created for six languages lacking VerbNet-like resources, including a tuned version of the Polish resource evaluated against the data and class membership criteria provided by five annotators and supplemented with additional verbs not considered in the pilot translation, can be readily employed for these purposes. On the theoretical side, our investigation provides empirical support for the long-standing linguistic hypothesis that Levin-style classification has cross-lingual potential. The cross-linguistic applicability of VerbNet-style classes is examined systematically based on a typologically varied sample of languages, which can inform further work in the area of cross-lingual transfer and creation of large-scale multilingual lexical resources.

These contributions open up many avenues for future work. Given the excellent results from human translation, it would be interesting to investigate whether machine translation could support at least some parts of the translation process. Since a degree of language-specific tuning seems inevitable, automatic translation could provide a starting point for humans who would perform the linguistic revision of the resource. Another possibility is to examine whether selection of candidate translations might be supported by semi-automatically produced resources such as the Predicate Matrix (Lacalle et al. 2016) and open WordNets (Bond and Foster 2013). The improved efficiency could facilitate creation of large-scale resources for a much larger set of languages and language families, including less-studied ones. Moreover, as discussed in Sect. 4.2, resources such as the Predicate Matrix can be utilised in conjunction with the manual method to help identify and increase coverage and precision of prototypical cases for the purposes of evaluation. Also, given the cross-lingual potential of VerbNet classification, the next natural step would be to use this type of classification to support multilingual NLP. Recent work on cross-lingual word embeddings has demonstrated that they can support cross-lingual projection methods (e.g., Guo et al. 2015; Ammar et al. 2016; Upadhyay et al. 2016; Vulić et al. 2017). An avenue worth pursuing would be to investigate how verb classes obtained via our linguistically informed translation method compare to more pragmatically driven Brown-style clusters, as in the work of Täckström et al. (2012) and Ammar et al. (2016)—such comparative study could shed more light on the usefulness of linguistically motivated approaches to the transfer of linguistic structure across languages. Finally, it would be interesting to extend the study presented in this paper to related resources such as WordNet, FrameNet and PropBank. This could yield deeper understanding of the properties of verbs that are translatable across languages.

## Electronic supplementary material

Below is the link to the electronic supplementary material.

Supplementary material 1 (pdf 77 KB)

Supplementary material 2 (pdf 229 KB)

## Footnotes

1 In the conative alternation, a transitive variant (*The girl kicked the ball*) alternates with a conative construction expressing an ‘attempted’ but not necessarily completed action in which the object of the verb is realised as the object of a prepositional phrase headed by ‘at’: *The girl kicked at the ball* (Levin 1993).

2 The translators were asked to list single words and phrases were not allowed (the only two-word items are reflexive verbs which in Croatian and Polish appear as verb + reflexive pronoun).

3 The pilot linguist was included so there were three linguists and three non-linguists.

4 Calculations were performed using the ReCal3 online application (Freelon 2010).

5 This issue has been observed in previous open-ended annotation tasks (McCarthy and Navigli 2007).

6 The resultative construction refers to a structure consisting of the main verb and a secondary predicate (an adjective or a prepositional phrase) which expresses the ‘result’, e.g., describes the state of the argument as a result of the action expressed by the verb: *Paul kicked the door* (basic transitive)—*Paul kicked the door open* (resultative).

7 In the causative-inchoative alternation, a construction including an unaccusative (inchoative) verb (*The snow melted, The vase broke*) alternates with a corresponding transitive construction, with the former unaccusative subject appearing in the direct object position (*The sun melted the snow, The cat broke the vase*).

8 The term ‘inchoative’ refers to the intransitive variant of a causatively alternating verb (i.e., a verb which can have a transitive and an intransitive meaning) describing a change of state undergone by the theme argument (e.g., *One of the bottles* *broke*) (see footnote 7).
