# Supplementary material for: Investigating the cross-lingual translatability of VerbNet-style classification
Source: Lang Resour Eval. 2017 Oct 20;52(3):771–99. doi: 10.1007/s10579-017-9403-x (PMC6428229; doi:10.1007/s10579-017-9403-x)
Supplement: Supplementary file 1 — Supplementary material 1 (pdf 77 KB) [file 10579_2017_9403_MOESM1_ESM.pdf]

Investigating the cross-lingual translatability of VerbNet-style classification

Language Resources and Evaluation

Olga Majewska (om304@cam.ac.uk), Ivan Vulić, Diana McCarthy, Yan Huang, Akira Murakami, Veronika Laippala, Anna Korhonen

Language Technology Lab (LTL), Department of Theoretical and Applied Linguistics (DTAL), University of Cambridge, 9 West Road, Cambridge CB3 9DP, UK

Online Resource 1 Guidelines for translation of verb classes

I Translation

1. Take one class and translate each of the 12 member verbs. For each member verb, its predominant sense (according to WordNet) should be translated. To check which sense that is, consult WordNet (http://wordnetweb.princeton.edu/perl/webwn) and pick the first (most frequent) sense of the verb. In a small minority of cases, the first sense in WordNet will not be appropriate, as it will not fit in semantically with the other class members. For example, the predominant sense of the verb 'beat' in WordNet is 'come out better in a competition, race, or conflict'. However, it is the sense listed below, 'hit repeatedly', which should be translated instead, as it involves a physical rather than metaphorical action. As a rule, the predominant sense in WordNet should be translated, unless it does not fit in the class - then the next relevant sense should be picked. The translated equivalent should be as close to the English definition as possible.
2. If several relevant translations are identified, each of them should be considered and kept for selection. To identify close synonyms, consult WordNet in the target language.
3. If an English verb lacks a direct equivalent in the target language, synonyms of already identified candidate verbs can be added, as in point 2. As a rule of thumb, if the number of candidate verbs in the target language is below 12, synonyms should be added, if possible. There is no upper limit, any number of relevant candidate verbs above 12 is fine (provided they closely convey the sense of the English verbs).

II Selection

4. The first stage of selection involves translation of syntactic frames and diathesis alternations found in VerbNet from English into the target language. To do that, access VerbNet online: http://verbs.colorado.edu/verb-index/search.php and find the class in question.
5. Consider the frames listed for the class (and its subclasses). Translate them into the target language, if possible. Syntactic structure, semantic roles, and selectional restrictions should be taken into account.
6. Having considered VerbNet frames, think of all other subcategorisation frames (and corresponding semantic roles) and diathesis alternations possible for a given candidate verb in the target language (valency dictionaries can be consulted, where available). These, together with those in point 5, will be used as selection criteria: verbs sharing frames and alternations will be kept in the class.
7. Taking one candidate verb at a time, from a given class, check if it can appear in the frames and alternations identified in 5 and 6. In most cases there will be a few frames and syntactic structures in which all or the majority of candidate verbs from a given class can appear. Those frames should be used as membership criteria for the class. Verbs which cannot appear in them should be eliminated.

8. Usually, if the majority of candidate verbs can appear in a certain frame or alternation, it should be considered as the basic frame for the class and used for selection. The minority of verbs which cannot appear in it should be eliminated. Sometimes, however, even if more than half of the candidate verbs appear in a certain frame, such frame should not be automatically taken as a criterion for eliminating the remaining verbs, if these share other basic frames, and therefore form a syntactically coherent class. The decision which frames to treat as basic frames for selection should be made individually for each class, based on native-speaker intuitions about whether verbs in question pattern together.
