# Supplementary material for: Investigating the cross-lingual translatability of VerbNet-style classification
Source: Lang Resour Eval. 2017 Oct 20;52(3):771–99. doi: 10.1007/s10579-017-9403-x (PMC6428229; doi:10.1007/s10579-017-9403-x)
Supplement: Supplementary file 2 — Supplementary material 2 (pdf 229 KB) [file 10579_2017_9403_MOESM2_ESM.pdf]

Investigating the cross-lingual translatability of VerbNet-style classification

Language Resources and Evaluation

Olga Majewska (om304@cam.ac.uk), Ivan Vulić, Diana McCarthy, Yan Huang, Akira Murakami, Veronika Laippala, Anna Korhonen

Language Technology Lab (LTL), Department of Theoretical and Applied Linguistics (DTAL), University of Cambridge, 9 West Road, Cambridge CB3 9DP, UK

Online Resource 2 Gold standard classes produced for 6 languages: Polish, Croatian, Mandarin, Japanese, Italian, and Finnish

Polish

VERBNET CLASS	VERBS
9.1 PUT	zakopać, pochować, pogrzebać, umiejscowić, umieścić, ulokować, instalować, montować, osadzać, położyć, ustawić, usytuować, składować, przechowywać, ukryć
10.1 REMOVE	usuwać, likwidować, obalić, zwolnić, wydalać, wydobyć, zrywać, eksmitować, wypędzać, wyrzucać, wycofać, odwoływać, odciąć, wytępić, wyplenić, wyjmować, wypuścić, wyrywać
11.1 SEND	wysyłać, transportować, nadawać, posyłać, przesyłać, transmitować, przenosić, przekazywać, dostarczać, przetaczać, transferować, przewozić, przeładować, doręczać
13.5.1 GET	wygrać, zdobyć, uzyskać, zarobić, kupić, pozyskać, przynosić, zbierać, wynająć, czarterować, wydzierżawić, zachować, nabyć
18.1 HIT	bić, trzepać, uderzać, walić, smagać, grzmocić, klepać, trzaskać, pukać, łomotać, palnąć, chłostać, rąbnąć, łupnąć, stukać, tłuc
22.2 AMALGAMATE	integrować, scalać, zbiegać się, porównywać, jednoczyć, stowarzyszać, zrzeszać, łączyć, korelować, unifikować, przeplatać, zamieniać się, pokrywać, harmonizować, konfrontować, współgrać, wiązać
29.2 CHARACTERIZE	wyobrażać sobie, przedstawiać, identyfikować, rozpoznawać, definiować, określać, prezentować, obrazować, diagnozować, precyzować
30.3 PEER	gapić się, patrzeć, spoglądać, zerkać, spozierać, zezować, wpatrywać się
31.1 AMUSE	stymulować, pobudzać, szokować, mylić, denerwować, przytłaczać, przerażać, przestraszyć, zawieść, rozczarować, zachwycać, wyczerpać, onieśmielać, zastraszyć, urzekać, wycieńczać, wykańczać, zadowalać, satysfakcjonować, poruszać
36.1 CORRESPOND	współpracować, współdziałać, kooperować, kolidować, kłócić się, zgadzać się, flirtować, romansować, spółkować, przekomarzać się, droczyć się, drażnić się, targować się, kojarzyć się, parzyć się
37.3 MANNER OF SPEAKING	krzyczeć, wrzeszczeć, szeptać, mruknąć, mamrotać, warczeć, jęczeć, zawodzić, chrząkać, piszczeć, jąkać, burknąć, wyć, wołać
37.7 SAY	mówić, odpowiadać, odrzekać, odpierać, wspominać, nadmieniać, napomknąć, oznajmić, donieść, ogłaszać, opowiadać, relacjonować, wykrzyknąć, odciąć się, zwierzać się, stwierdzać, komunikować, oświadczać, rzec, wyznać, wyjawiać
40.2 NONVERBAL EXPRESSION	uśmiechać się, śmiać się, szczerzyć się, wzdychać, sapać, chichotać, szydzić, drwić, naśmiewać się, ziewać, szlochać, łkać, chrapać, chlipać, zipać
43.1 LIGHT EMISSION	świecić, błyszczeć, błyskać, płonąć, jarzyć się, mienić się, promienieć, migotać, lśnić, skrzyć się, żarzyć się, migać, rozbłysnąć, tlić się, buchać, gorzeć, szklić się
45.4 CHANGE OF STATE	wzmocnić, zwęzić, osłabić, ścieśnić, pogłębić, topić, mnożyć, powielać, wyostrzyć, skrócić, poszerzyć, rozszerzyć, rozpuścić
47.3 MODES OF BEING WITH MOTION	trząść, chwiać, kołysać, bujać, łopotać, trzepotać, furkotać, wirować, chybotać, podrygiwać, poruszać, pulsować, tętnić, wibrować, telepać, wahać, huśtać
51.3.2 RUN	pływać, latać, fruwać, chodzić, ślizgać się, biegać, podróżować, spacerować, przechadzać się, sunąć, szybować, maszerować, truchtać, kłusować, cwałować, galopować

Croatian

VERBNET CLASS	VERBS
9.1 PUT	pokopati, zakopati, staviti, postaviti, deponirati, pozicionirati, namjestiti, uroniti, umetnuti, pohraniti, smjestiti, metnuti
10.1 REMOVE	ukloniti, otkloniti, ukinuti, istovariti, izbaciti, izvući, izvaditi, oduzeti, oduzimati, iskorijeniti, odvojiti, istjerati, uvući, izbrisati
11.1 SEND	slati, poslati, otpremiti, odašiljati, prenijeti, prebaciti, dostaviti, tutnuti, prevoziti, transportirati, proslijediti, dopremiti, uputiti
13.5.1 GET	dobiti, osvojiti, steći, zaraditi, kupiti, nabaviti, rezervirati, donijeti, dohvatiti, dostići, odabrati, izabrati, unajmiti, zakupiti, čuvati, sačuvati
18.1 HIT	tući, udariti, pljusnuti, ošamariti, šamarati, lupiti, batinati, zabiti, nabiti, prikucati, išibati, bičevati, tresnuti, zveknuti
22.2 AMALGAMATE	spojiti, suprotstaviti, razlikovati se, odgovarati, slagati se, preklapati, preplitati, ujediniti, sjediniti, udruživati, pripojiti, uključiti,integrirati, podudarati se, inkorporirati, porediti, usporediti, korelirati, alternirati, izmijenjivati se
29.2 CHARACTERIZE	razmotriti, predočiti, portretirati, oslikati, smatrati, razmotriti, regrutirati, angažirati, odrediti, definirati, opisati, dijagnosticirati, identificirati, postaviti, predstaviti, klasificirati
30.3 PEER	slušati, poslušati, buljiti, zuriti, gledati, pogledati, piljiti, motriti, viriti, škiljiti, njuškati, zjapiti, zagledati, proviriti, zaviriti
31.1 AMUSE	uživati, očarati, uplašiti, šokirati, zaprepastiti, zbuniti, uznemiriti, uzrujati, pobijediti, nadjačati, razočarati, iscrpiti, zastrašiti, zaplašiti, preplašiti, stimulirati, potaknuti, prijetiti, zaprijetiti
36.1 CORRESPOND	surađivati, sudariti se, složiti se, podudarati se, pariti se, spariti, flertati, koketirati, djelovati, razilaziti se, nagoditi se, podsmjehivati se, cjenkati se, suosjećati, odbjeći
37.3 MANNER OF SPEAKING	vikati, vrištati, stenjati, jaukati, mrmljati, žagoriti, režati, naricati, šaptati, šaputati, gunđati, roktati, groktati, cviljeti, mucati
37.7 SAY	reći, kazati, odgovoriti, uzvratiti, spomenuti, iznijeti, iskazati, izvijestiti, najaviti, obavijestiti, obznaniti, ispričati, izustiti, izjaviti, uzviknuti, uzvratiti, povjeriti
40.2 NONVERBAL EXPRESSION	osmjehnuti se, smješkati se, smijati se, nasmijati se, ceriti se, kesiti se, uzdahnuti, cerekati se, mrštiti se, namrštiti se, hihotati se, dahtati, podsmjehnuti se, zijevati, jecati, ridati, hrkati
43.1 LIGHT EMISSION	sijati, zasijati, bljesnuti, buknuti, planuti, plamtjeti, svjetlucati, treperiti, presijavati se, blještati, blistati, gorjeti
45.4 CHANGE OF STATE	ublažiti, smekšati, oslabiti, slabiti, suziti, produbiti, vlažiti, ovlažiti, namočiti, rastopiti, otopiti, rastaliti, topiti, umnožiti, namnožiti, ojačati, učvrstiti, osnažiti, izoštriti, naoštriti, zašiljiti, skratiti, proširiti, raširiti, zamrznuti, smrznuti, zalediti
47.3 MODES OF BEING WITH MOTION	tresti se, drhtati, posrnuti, njihati, lelujati, vitlati, kovitlati se, klackati se, lepršati, vijoriti se, viti, klimati se, lebdjeti, strujati, treperiti, drhtati, mrdati se, meškoljiti se, koprcati se, vibrirati, titrati, oscilirati
51.3.2 RUN	plivati, letjeti, hodati, kliznuti, skliznuti, trčati, putovati, šetati, kliziti, trčati, marširati, stupati, kasati, kaskati, galopirati

Mandarin

VERBNET CLASS	VERBS
9.1 PUT	遮盖，掩藏，放，安装，安放，存放，放置，摆，定位，浸，插入，嵌入，贮藏
10.1 REMOVE	除掉，废除，完成，释放，驱逐，拔出，扣除，根除，切断，减去，撤销，收回，删除
11.1 SEND	运送，邮寄，投递，发送，寄，传递，传达，转移，送交，运输，悄放，运输，输送，派遣，转寄，调
13.5.1 GET	赢得，获取，挣，赚得，买，得到，拿来，预订，预约，取来，获得，达到，采，包租，保存
18.1 HIT	揍打，拍打，撞击，敲打，重敲，猛击，捶打，鞭打，痛击，猛击，掴，拍击，重打，撞，碰
22.2 AMALGAMATE	对比，配对，重叠，联合，结合，统一，结交，结伴，合并，同时发生，比较，相关，交替，轮换
29.2 CHARACTERIZE	设想，想象，描绘，看待，认为，视，当,定义，阐述，描述，诊断，认出，识别，归类
30.3 PEER	听，凝视，看，瞥，扫视，注视，窥视，偷看，眯眼看，斜看，窥探，瞪眼看，斜看，窥探
31.1 AMUSE	取悦，惊吓，震惊，困惑，烦扰，笼罩，覆盖，恫吓，恐吓，吓唬，激发，刺激，威胁，危及
36.1 CORRESPOND	合作，抵触，对立，同意，交配，挑逗，互动，不同意，妥协，退让，逗弄，讨价还价，同情，怜悯，私奔，出走
37.3 MANNER OF SPEAKING	大叫，喊，叫喊，呻吟，呜咽，咕哝，低语，怒吼，哀号，悲叹，私语，耳语，含糊地说，抽咽，结巴地说
37.7 SAY	说，回复，回答，提到，说明，陈述，报道，报告，答复，宣布，描述，详述，说出，发出，呼喊，惊叫，反驳，吐露
40.2 NONVERBAL EXPRESSION	微笑，笑，咧嘴笑，叹气，咯咯笑，皱眉，傻笑，喘气，冷笑，打呵欠，啜泣，打鼾
43.1 LIGHT EMISSION	反光，发光，闪光，闪耀，放光，发白热光，发亮，闪烁，发微光，闪闪发光，照耀，变亮
45.4 CHANGE OF STATE	软化，柔化，削弱，收窄，加深，弄湿，融化，增加，强化，巩固，磨锋，缩短，减少，拓宽，冰冻
47.3 MODES OF BEING WITH MOTION	震动，摇晃，蹒跚，摇动，摆动，旋动，踉跄，晃动，飘荡，漂浮，颤抖，战栗，摇摆，颤动，振荡
51.3.2 RUN	游泳，飞，走，滑，跑，旅行，漫步，闲逛，滑行，慢跑，行军，小跑，飞跑，疾驰

Japanese

VERBNET CLASS	VERBS
9.1 PUT	埋める、置く、設置する、取り付ける、位置させる、浸す、入れる、隠す
10.1 REMOVE	取り除く、止める、取り出す、抜き出す、差し引く、根絶する、切断する、立ち退かせる、撤回する、削除する
11.1 SEND	送る、 伝える、移す、配達する、滑りこませる、運ぶ、送付する、転送する
13.5.1 GET	得る、買う、予約する、拾う、借りる
18.1 HIT	はたく、ノックする、強打する、ぶつける
22.2 AMALGAMATE	合わせる、重ねる、結合する、統合する、一致する、匹敵する、相関する、交代させる
29.2 CHARACTERIZE	想定する、見なす、扱う、定義する、診断する、特定する、分類する
30.3 PEER	聞く、見つめる、見る、ぱっと見る、流し目を送る、覗き見る
31.1 AMUSE	歓喜させる、怖がらせる、困惑させる、動揺させる、圧倒する、がっかりさせる、疲弊させる、脅す、刺激する
36.1 CORRESPOND	協力する、衝突する、同意する、つがう、 交流する、妥協する、冷やかす、値切る、同情する、駆け落ちする
37.3 MANNER OF SPEAKING	叫ぶ、嘆く、つぶやく、うなる、ささやく
37.7 SAY	言う、返答する、述べる、報告する、応答する、発表する、叫ぶ、言い返す、打ち明ける
40.2 NONVERBAL EXPRESSION	にこりとする、笑う、溜息をつく、くすくす笑う、顔をしかめる、あえぐ、あざ笑う、あくびをする、すすり泣く、いびきをかく
43.1 LIGHT EMISSION	輝く、閃く、燃える、きらめく、光る
45.4 CHANGE OF STATE	和らげる、弱める、狭める、深める、湿らせる、溶かす、増やす、強化する、研ぐ、縮める、広げる、凍らせる
47.3 MODES OF BEING WITH MOTION	揺れる、渦巻く、ぐらつく、漂う、震わせる、振動する
51.3.2 RUN	泳ぐ、飛ぶ、歩く、滑る、走る、移動する、散歩する、ジョギングする、行進する

Italian

VERBNET CLASS	VERBS
9.1 PUT	collocare, installare, montare, mettere, depositare, posizionare, situare, immergere, inserire
10.1 REMOVE	rimuovere, togliere, abolire, rilasciare, espellere, estrarre, eradicare, staccare, sgombrare, sottrarre, ritrarre, eliminare
11.1 SEND	spedire, inviare, mandare, trasmettere, trasferire, consegnare, trasportare, inoltrare
13.5.1 GET	vincere, guadagnare, comprare, acquistare, ottenere, prenotare, riservare, prendere, raggiungere, raccogliere, noleggiare, conservare
18.1 HIT	picchiare, battere, percuotere, schiaffeggiare, colpire, martellare, frustare, sculacciare, sbattere, urtare
22.2 AMALGAMATE	contrastare, sovrapporre, unire, affiliare, integrare, incorporare, paragonare, correlare, alternare
29.2 CHARACTERIZE	prevedere, ritrarre, raffigurare, riguardare, trattare, elencare, definire, diagnosticare, identificare, riconoscere, ristabilire, classificare
30.3 PEER	ascoltare, fissare, osservare, scrutare, guardare, sbirciare, spiare
31.1 AMUSE	dilettare, impaurire, scioccare, confondere, turbare, sconvolgere, sovrastare, deludere, esaurire, intimidire, spaventare, stimolare, minacciare
36.1 CORRESPOND	cooperare, collaborare, scontrarsi, concorrere, accoppiarsi, flirtare, interagire, dissentire
37.3 MANNER OF SPEAKING	urlare, gridare, strillare, biascicare, mormorare, ringhiare, sussurrare, brontolare, grugnire, balbettare
37.7 SAY	dire, menzionare, affermare, dichiarare, riportare, annunciare, raccontare, narrare, relazionare, riferire, pronunciare, esclamare, confidare
40.2 NONVERBAL EXPRESSION	sorridere, ridere, digrignare, sospirare, ridacchiare, ansimare, sogghignare, sbadigliare, singhiozzare, russare
43.1 LIGHT EMISSION	brillare, luccicare, risplendere, fiammeggiare, baluginare, scintillare, splendere, sfavillare
45.4 CHANGE OF STATE	ammorbidire, indebolire, limitare, restringere, approfondire, inumidire, moltiplicare, sciogliere, rafforzare, appuntire, accorciare, ampliare, congelare
47.3 MODES OF BEING WITH MOTION	tremare, scuotersi, vacillare, ondeggiare, mulinare, vorticare, traballare, barcollare, svolazzare, diffondersi, tremare, fremere, oscillare
51.3.2 RUN	nuotare, volare, camminare, scivolare, correre, viaggiare, passeggiare, marciare, trottare, galoppare

Finnish

VERBNET CLASS	VERBS
9.1 PUT	asettaa, asentaa, kiinnittää, laittaa, panna, kätkeä
10.1 REMOVE	poistaa, lakkauttaa, vapauttaa, vähentää, tuhota, hävittää, katkaista, häätää, vähentää, vetää, perua
11.1 SEND	lähettää, postittaa, toimittaa, siirtää, kuljettaa, välittää
13.5.1 GET	ansaita, ostaa, varata, hakea, noutaa, vuokrata
18.1 HIT	lyödä, hakata, nuijia
22.2 AMALGAMATE	yhdistää, liittää
29.2 CHARACTERIZE	nähdä, esittää, kuvailla, määrittää, kuvata
30.3 PEER	kuunnella, tuijottaa, katsella, vilkaista, silmäillä, tuijottaa, tiirailla, tähyillä, kurkkia, kurkata, vilkuilla, töllistellä, mulkaista, tirkistellä
31.1 AMUSE	ilahduttaa, riemastuttaa, ilostuttaa, pelottaa, pelästyttää, pelotella, säikäyttää, säikäytellä, järkyttää, shokeerata, hämätä, hämmentää, järkyttää, hermostuttaa, häkellyttää, väsyttää, uuvuttaa, hiljentää, innostaa, yllyttää, vaientaa, häkeltää, murskata, uhata, vaarantaa, uhkailla
36.1 CORRESPOND	pilailla, tinkiä
37.3 MANNER OF SPEAKING	huutaa, karjua, kiljua, mölistä, ähkiä, huokailla, vaikeroida, voihkia, nurista, mumista, supista, mutista, murista, äristä, ärjähtää, ulvoa, kuiskata, röhkiä, murahtaa, röhkäistä, vikistä, uikuttaa, ulista, änkyttää
37.7 SAY	huutaa, huudahtaa, sanoa, mainita, vastata, ilmoittaa, julistaa, kertoa, selostaa, todeta, raportoida
40.2 NONVERBAL EXPRESSION	hymyillä, nauraa, irvistää, virnistää, henkäistä, huokaista, huoata, huokailla, myhäillä, hykertää, kurtistaa, kikattaa, huohottaa, läähättää, haukotella, nyyhkyttää, kuorsata
43.1 LIGHT EMISSION	loistaa, hohtaa, välkkyä, välähtää, vilkkua, roihuta, loimuta, hehkua, paistaa, liekehtiä, kimaltaa, lepattaa, välkehtiä, kipinöidä, kimmeltää, tuikkia, kimallella, leiskua
45.4 CHANGE OF STATE	kaventaa, syventää, kostuttaa, teroittaa, lyhentää, laajentaa, laventaa, vaimentaa, heikentää, vahventaa
47.3 MODES OF BEING WITH MOTION	tutista, hyllyä, heilua, aaltoilla, heilahdella, täristä, värähdellä, horjua, kieppua, väristä, järistä, järähdellä, keinua, keinahdella, huojahdella, kiertyä, horjua, hoippua, hulmuta, liehua, vaappua, tuulahtaa, vavista, hytistä
51.3.2 RUN	juosta, matkustaa, käveleskellä, kuljeskella, kuljeksia, käppäillä, liukua, lipua, liitää, marssia, ravata, jolkutella, laukata, nelistää
